# Internal capsule microstructure mediates the relationship between childhood maltreatment and PTSD following adulthood trauma exposure

**DOI:** 10.1038/s41380-023-02012-3

**Published:** 2023-03-17

**Authors:** Samantha A. Wong, Lauren A. M. Lebois, Timothy D. Ely, Sanne J. H. van Rooij, Steven E. Bruce, Vishnu P. Murty, Tanja Jovanovic, Stacey L. House, Francesca L. Beaudoin, Xinming An, Donglin Zeng, Thomas C. Neylan, Gari D. Clifford, Sarah D. Linnstaedt, Laura T. Germine, Kenneth A. Bollen, Scott L. Rauch, John P. Haran, Alan B. Storrow, Christopher Lewandowski, Paul I. Musey, Phyllis L. Hendry, Sophia Sheikh, Christopher W. Jones, Brittany E. Punches, Michael C. Kurz, Robert A. Swor, Lauren A. Hudak, Jose L. Pascual, Mark J. Seamon, Claire Pearson, David A. Peak, Roland C. Merchant, Robert M. Domeier, Niels K. Rathlev, Brian J. O’Neil, Paulina Sergot, Leon D. Sanchez, Mark W. Miller, Robert H. Pietrzak, Jutta Joormann, Deanna M. Barch, Diego A. Pizzagalli, Steven E. Harte, James M. Elliott, Ronald C. Kessler, Karestan C. Koenen, Samuel A. McLean, Kerry J. Ressler, Jennifer S. Stevens, Nathaniel G. Harnett

**Affiliations:** 1 Division of Depression and Anxiety, McLean Hospital, Belmont, MA USA; 2 Department of Psychiatry, Harvard Medical School, Boston, MA USA; 3 Department of Psychiatry and Behavioral Sciences, Emory University School of Medicine, Atlanta, GA USA; 4 Department of Psychological Sciences, University of Missouri—St. Louis, St. Louis, MO USA; 5 Department of Psychology, Temple University, Philadelphia, PA USA; 6 Department of Psychiatry and Behavioral Neurosciences, Wayne State University, Detroit, MI USA; 7 Department of Emergency Medicine, Washington University School of Medicine, St. Louis, MO USA; 8 Department of Epidemiology, Brown University, Providence, RI USA; 9 Department of Emergency Medicine, Brown University, Providence, RI USA; 10 Institute for Trauma Recovery, Department of Anesthesiology, University of North Carolina at Chapel Hill, Chapel Hill, NC USA; 11 Department of Biostatistics, Gillings School of Global Public Health, University of North Carolina, Chapel Hill, NC USA; 12 Departments of Psychiatry and Neurology, University of California San Francisco, San Francisco, CA USA; 13 Department of Biomedical Informatics, Emory University School of Medicine, Atlanta, GA USA; 14 Department of Biomedical Engineering, Georgia Institute of Technology and Emory University, Atlanta, GA USA; 15 Institute for Technology in Psychiatry, McLean Hospital, Belmont, MA USA; 16 The Many Brains Project, Belmont, MA USA; 17 Department of Psychology and Neuroscience & Department of Sociology, University of North Carolina at Chapel Hill, Chapel Hill, NC USA; 18 Department of Psychiatry, McLean Hospital, Belmont, MA USA; 19 Department of Emergency Medicine, University of Massachusetts Chan Medical School, Worcester, MA USA; 20 Department of Emergency Medicine, Vanderbilt University Medical Center, Nashville, TN USA; 21 Department of Emergency Medicine, Henry Ford Health System, Detroit, MI USA; 22 Department of Emergency Medicine, Indiana University School of Medicine, Indianapolis, IN USA; 23 Department of Emergency Medicine, University of Florida College of Medicine—Jacksonville, Jacksonville, FL USA; 24 Department of Emergency Medicine, Cooper Medical School of Rowan University, Camden, NJ USA; 25 Department of Emergency Medicine, Ohio State University College of Medicine, Columbus, OH USA; 26 Ohio State University College of Nursing, Columbus, OH USA; 27 Department of Emergency Medicine, University of Alabama School of Medicine, Birmingham, AL USA; 28 Department of Surgery, Division of Acute Care Surgery, University of Alabama School of Medicine, Birmingham, AL USA; 29 Center for Injury Science, University of Alabama at Birmingham, Birmingham, AL USA; 30 Department of Emergency Medicine, Oakland University William Beaumont School of Medicine, Rochester, MI USA; 31 Department of Emergency Medicine, Emory University School of Medicine, Atlanta, GA USA; 32 Department of Surgery, Department of Neurosurgery, University of Pennsylvania, Philadelphia, PA USA; 33 Perelman School of Medicine, University of Pennsylvania, Philadelphia, PA USA; 34 Department of Surgery, Division of Traumatology, Surgical Critical Care and Emergency Surgery, University of Pennsylvania, Philadelphia, PA USA; 35 Department of Emergency Medicine, Wayne State University, Ascension St. John Hospital, Detroit, MI USA; 36 Department of Emergency Medicine, Massachusetts General Hospital, Boston, MA USA; 37 Department of Emergency Medicine, Brigham and Women’s Hospital, Boston, MA USA; 38 Department of Emergency Medicine, Saint Joseph Mercy Hospital, Ypsilanti, MI USA; 39 Department of Emergency Medicine, University of Massachusetts Medical School-Baystate, Springfield, MA USA; 40 Department of Emergency Medicine, Wayne State University, Detroit Receiving Hospital, Detroit, MI USA; 41 Department of Emergency Medicine, McGovern Medical School at UTHealth, Houston, TX USA; 42 Department of Emergency Medicine, Harvard Medical School, Boston, MA USA; 43 National Center for PTSD, Behavioral Science Division, VA Boston Healthcare System, Boston, MA USA; 44 Department of Psychiatry, Boston University School of Medicine, Boston, MA USA; 45 National Center for PTSD, Clinical Neurosciences Division, VA Connecticut Healthcare System, West Haven, CT USA; 46 Department of Psychiatry, Yale School of Medicine, New Haven, CT USA; 47 Department of Psychology, Yale University, New Haven, CT USA; 48 Department of Psychological & Brain Sciences, Washington University in St. Louis, St. Louis, MO USA; 49 Department of Anesthesiology, University of Michigan Medical School, Ann Arbor, MI USA; 50 Department of Internal Medicine-Rheumatology, University of Michigan Medical School, Ann Arbor, MI USA; 51 Kolling Institute, University of Sydney, St Leonards, NSW Australia; 52 Faculty of Medicine and Health, University of Sydney, Northern Sydney Local Health District, Camperdown, NSW Australia; 53 Physical Therapy & Human Movement Sciences, Feinberg School of Medicine, Northwestern University, Chicago, IL USA; 54 Department of Health Care Policy, Harvard Medical School, Boston, MA USA; 55 Department of Epidemiology, Harvard T.H. Chan School of Public Health, Harvard University, Boston, MA USA; 56 Department of Emergency Medicine, University of North Carolina at Chapel Hill, Chapel Hill, NC USA; 57 Institute for Trauma Recovery, Department of Psychiatry, University of North Carolina at Chapel Hill, Chapel Hill, NC USA

**Keywords:** Prognostic markers, Psychiatric disorders, Psychology, Neuroscience

## Abstract

Childhood trauma is a known risk factor for trauma and stress-related disorders in adulthood. However, limited research has investigated the impact of childhood trauma on brain structure linked to later posttraumatic dysfunction. We investigated the effect of childhood trauma on white matter microstructure after recent trauma and its relationship with future posttraumatic dysfunction among trauma-exposed adult participants (*n* = 202) recruited from emergency departments as part of the AURORA Study. Participants completed self-report scales assessing prior childhood maltreatment within 2-weeks in addition to assessments of PTSD, depression, anxiety, and dissociation symptoms within 6-months of their traumatic event. Fractional anisotropy (FA) obtained from diffusion tensor imaging (DTI) collected at 2-weeks and 6-months was used to index white matter microstructure. Childhood maltreatment load predicted 6-month PTSD symptoms (b = 1.75, SE = 0.78, 95% CI = [0.20, 3.29]) and inversely varied with FA in the bilateral internal capsule (IC) at 2-weeks (*p* = 0.0294, FDR corrected) and 6-months (*p* = 0.0238, FDR corrected). We observed a significant indirect effect of childhood maltreatment load on 6-month PTSD symptoms through 2-week IC microstructure (b = 0.37, Boot SE = 0.18, 95% CI = [0.05, 0.76]) that fully mediated the effect of childhood maltreatment load on PCL-5 scores (b = 1.37, SE = 0.79, 95% CI = [−0.18, 2.93]). IC microstructure did not mediate relationships between childhood maltreatment and depressive, anxiety, or dissociative symptomatology. Our findings suggest a unique role for IC microstructure as a stable neural pathway between childhood trauma and future PTSD symptoms following recent trauma. Notably, our work did not support roles of white matter tracts previously found to vary with PTSD symptoms and childhood trauma exposure, including the cingulum bundle, uncinate fasciculus, and corpus callosum. Given the IC contains sensory fibers linked to perception and motor control, childhood maltreatment might impact the neural circuits that relay and process threat-related inputs and responses to trauma.

## Introduction

Childhood trauma is a well-established risk factor for development of trauma and stress-related disorders in adulthood. Early life stress may interact with stressors in adulthood to increase an individual’s risk for posttraumatic stress disorder (PTSD), major depression, substance use, or behavioral disorders [1]. Furthermore, childhood trauma is associated with variability in brain circuits known to play a role in PTSD, which could represent potential neural signatures of PTSD susceptibility. However, limited work to date has investigated neural correlates of how earlier childhood trauma augments posttraumatic reactions after a trauma sustained as an adult. Identifying the neurobiological correlates of childhood trauma related risk for acute stress reactions in adulthood may advance neuroscience-based approaches for prediction and prevention of PTSD development.

PTSD is thought to be partially driven by dysfunction of threat learning neurocircuitry – particularly the prefrontal cortex, hippocampus, and amygdala – as a result of a traumatic experience [2–4]. White matter tracts such as the cingulum bundle, uncinate fasciculus, and fornix/stria terminalis interconnect threat neurocircuitry regions and are thought to be involved in PTSD-related dysfunction (See [5] for review), potentially due to experience-dependent changes in tract microstructure [6]. In line with this reasoning, previous PTSD research has investigated Fractional Anisotropy (FA) as one of several measures to index white matter microstructure derived from Diffusion Tensor Imaging (DTI). Greater FA indicates greater linearity in the flow of water molecules due to constraint by myelinated tracts. Individuals with PTSD show reduced FA of the cingulum bundle and uncinate fasciculus [7–11], which interconnects the prefrontal cortex, amygdala, and hippocampus, although there is some heterogeneity in findings [12, 13]. Successful psychotherapy for PTSD appears to lead to increased FA in tracts such as the cingulum and fornix [14]. Further, studies of recent trauma exposure suggest variability in these same tracts are related to future development of PTSD such that lower FA is generally related to greater PTSD symptom severity [15–18]. Taken together, the previous work suggests white matter tracts of core threat neurocircuitry are related to the development and expression of PTSD symptoms.

Despite the importance of threat neurocircuitry white matter tracts, emergent research in childhood and adult trauma suggests that PTSD-related white matter alterations may additionally occur within other tracts [19–21]. Previous retrospective and meta-analytic DTI studies demonstrate that childhood trauma exposure is associated with alterations in FA both within threat neurocircuitry tracts and sensory integration tracts such as the anterior thalamic radiation, superior longitudinal fasciculus, inferior fronto-occipital fasciculus, optic radiations, and arcuate fasciculus [20–27]. Further, recent meta-analyses from the PGC-PTSD and ENIGMA groups found that the largest reduction in FA for individuals with PTSD was not within threat neurocircuitry tracts, but instead within the tapetum of the corpus callosum [28]. Perception and integration of sensory stimuli is necessary for appropriate threat learning [29]. The prior findings thus suggest trauma and PTSD-related FA reductions may extend outside threat neurocircuitry and encompass regions necessary for stimulus perception.

Limited research exists on the interrelationship between childhood trauma, white matter microstructure, and posttraumatic outcomes following a more recent trauma, though it may improve our understanding of the biological basis of PTSD. However, previous studies have found relationships between childhood trauma, brain structure, and stressors in adulthood [30–33]. In one study, total childhood trauma exposure moderated the effect of later combat exposure on FA within the hippocampal component of the cingulum, with greater childhood trauma and combat exposure related to decreased FA [30]. In a longitudinal study of young adults, uncinate fasciculus FA values at baseline moderated the relationship between recent stressors (e.g., break up with romantic partner, failing a course, or financial problems) and mood and anxiety symptoms at follow up among those with higher reported childhood maltreatment [31]. Limited work, however, has considered potential associations with white matter tracts outside threat neurocircuitry, which may be important in light of recent findings of PTSD-related FA reductions.

The present study investigated whether, among recent trauma survivors, brain white matter microstructure mediated the effect of childhood maltreatment exposure on posttraumatic dysfunction. Given prior findings in both studies on threat neurocircuitry of PTSD and emergent work implicating sensory and other white matter tracts, we assessed FA across white matter tracts using a whole-brain approach following previous work by the PGC-ENIGMA consortium [28]. We hypothesized that white matter FA at 2 weeks post-trauma, in general, would be negatively associated with childhood maltreatment load. We further hypothesized that white matter FA associated with childhood maltreatment would mediate associations between childhood maltreatment and posttraumatic outcomes after a recent trauma. Our findings highlight a neural pathway through which childhood trauma may confer risk for acute stress reactions in adulthood and shed light on white matter markers of susceptibility for PTSD.

## Materials and methods

### Participants

Participants were recruited as part of the AURORA study, a longitudinal multisite investigation of adverse neuropsychiatric sequalae [34]. Participants included in this investigation have been reported on in previous work [35–38]. However, the investigation described here is the first to consider the relationships of childhood maltreatment exposure, white matter microstructure, and later posttraumatic outcomes. As detailed in our prior reports [34], enrollment occurred at emergency departments (ED) and focused on those presenting within the 72 h following exposure to a qualifying trauma (physical or sexual assault, motor vehicle accident, fall >10 feet, mass casualty incident, or other life-threatening traumatic event reported on a screener question and agreed upon as a plausible qualifying event by the study staff). Participants were included if they were English-speaking, between 18 and 75 years-old, and able to consent and follow study procedures. Participants were recruited regardless of prior PTSD symptoms or diagnosis and were asked to report retrospectively on prior PTSD (and other disorders) symptoms in the emergency department. General exclusion criteria for the AURORA study have been described previously [34]. MRI collection exclusion criteria were having metal or ferromagnetic implants, history of seizure or epilepsy, history of Parkinson’s disease, dementia, or Alzheimer’s disease, current pregnancy, and/or declining to complete the MRI. From the beginning of study enrollment in September 2017 to July 2020, MRI data were collected within ~2 weeks of trauma exposure for 439 participants and DTI data were available from 353 participants. Participants were excluded for MRI quality issues (*n* = 37) (e.g., motion artefact, anatomical barriers, or low-quality data). The present analyses focused on participants who completed both DTI and the abbreviated Childhood Trauma Questionnaire (described below) at 2-weeks and posttraumatic outcome measures at 6-months post qualifying trauma and excluded participants missing a required questionnaire (*n* = 153). A total of 202 participants were retained for final analyses. Further analyses of DTI data from a subset of 85 participants (*n* = 111 collected, *n* = 26 excluded) collected at a 6-month follow-up imaging session also were completed. All participants provided informed consent as approved by the Biomedical IRB at UNC Chapel Hill through the office of Human Research Ethics, the central IRB for all study sites.

### Baseline surveys and socio-demographics

Participants completed a baseline assessment in the ED that included self-reported trauma characteristics and demographic characteristics [34]. Age, sex assigned at birth, race/ethnicity, highest education level, marital status, employment status, and total household income were obtained in the ED baseline surveys. Patients were also asked if they hit their head or experienced a head injury during the event that brought them to the ED (*n* = 86 endorsed).

### Childhood maltreatment load

An abbreviated 11-item version of the Childhood Trauma Questionnaire—Short Form (CTQ-SF; [39, 40]) was used to index childhood maltreatment. Items were selected from the CTQ-SF to capture maltreatment subscales while minimizing participant burden (individual questions selected provided in the supplementary information). Items selected to capture childhood maltreatment showed high internal reliability (Cronbach’s a = 0.92). The questionnaire was administered two weeks after the qualifying trauma. Items were self-reported on a 5-point Likert scale (0: never, 1: rarely, 2: sometimes, 3: often, 4: very often). The maltreatment subtypes evaluated include emotional abuse (sub-score range: 0 to 8), physical abuse (sub-score range: 0 to 8), sexual abuse (sub-score range: 0 to 12), emotional neglect (sub-score range: 0 to 8), and physical neglect (sub-score range: 0 to 8). Total possible summed scores ranged from 0 to 44. We indexed childhood maltreatment load as the endorsements of moderate to extreme levels of each maltreatment subtype [41–43]. Moderate to extreme abuse or neglect for other maltreatment subtypes were defined as a subtype score of 4 or above. Moderate to extreme sexual abuse was defined as a sub-score of 3 or above on sexual abuse items. These cutoffs were modified for the abbreviated assessment from clinical cutoffs previously suggested [39]. The sum of moderate to extreme maltreatment types was used to index total childhood maltreatment load.

### Lifetime trauma

Lifetime trauma exposure was assessed with the Life Events Checklist (LEC-5), an established 17-item instrument assessing exposure to 17 traumatic life events [44, 45]. Participants completed the LEC-5 at 8-weeks after their qualifying exposure. Participants indicated if a selection of traumatic experiences happened to them personally, if they witnessed it happen to someone else, learned about it happening to someone close to them, or was exposed to details about it as part of their job. A modified total LEC score (mLEC-5, range: 0 to 17) was calculated by summing the types of traumatic events endorsed, regardless of exposure modality. Although participants could endorse experiencing a life event in multiple ways (e.g., “happened to me,” “witnessed it”), any exposure to a given traumatic life event resulted in the maximal score of one for the modified total LEC score.

### Posttraumatic outcomes

Posttraumatic dysfunction was assessed in terms of PTSD, depression, anxiety, and dissociation symptoms at 6-months following the index trauma. PTSD symptoms were assessed using the Posttraumatic Stress Disorder (PTSD) checklist for DSM-5 (PCL-5), a psychometrically rigorous 20-item questionnaire on symptom presence and severity [46, 47]. Participants rated symptom severity on a scale of 0 (not at all) to 4 (extremely). Depression symptoms were assessed with the 8-item Patient-Reported Outcomes Measurement Information System (PROMIS) Depression instrument, short form 8b [48, 49]. A total raw score was computed from summing the individual items and then converted to a T-score [50]. Anxiety symptoms were assessed with 4-items from the PROMIS Anxiety bank [49, 51]. Participants rated how often they felt tense, worried about things, had trouble relaxing, or felt anxious on a scale of 1 (none of the time) to 5 (all or almost all of the time), and item scores were summed to create a total anxiety score. Dissociation was assessed using a modified 2-item Brief Dissociative Experiences Scale (DES-B-Modified, [52]). Participants rated how often they felt people, objects, or the world around them seemed unreal, and how often they felt they were looking through a fog so that people and things seemed unclear on a scale from 1 (none of the time) to 5 (all or almost all of the time). A sum of the two questions was used as an index of dissociation severity.

### Diffusion tensor imaging

Diffusion weighted imaging (DWI) data were collected across five sites (Table S1). Data processing was similar to prior reports [36, 53], following the recommendations of the ENIGMA consortium (http://enigma.ini.usc.edu/protocols/dti-protocols/). To ensure quality data, raw data were visually inspected, and we calculated metrics of temporal signal-to-noise ratio and outlier maximum voxel intensity as in a prior report [54]. Participants who demonstrated both: (a) TSNR values lower than 4.88 and (b) maximum voxel intensities greater than 5000 were removed from analyses to retain the maximum number of participants while removing low-quality data. Briefly, motion and eddy current effects in the DWI data were reduced using the ‘eddy’ subroutine in FSL and susceptibility effects were corrected for using nonlinear warping of the DWI data to the participant’s T1-weighted anatomical scan [55–57]. Tract-Based Spatial Statistics (TBSS) processing was used as implemented in the ENIGMA-DTI working group processing standards to extract FA values across white matter regions [58, 59]. First, FA maps were non-linearly registered to the standard ENIGMA FA map in Montreal Neurological Institute (MNI) standard space [59]. The ENIGMA FA skeleton map was then projected onto each subjects FA maps in standard space. Finally, regional FA values were extracted from the John’s Hopkins University (JHU) White matter atlas [60] and used in group level analyses. We also extracted axial diffusivity (AD), radial diffusivity (RD) and mean diffusivity (MD) for exploratory follow-up analyses (see Supplementary Information).

### Statistical analysis

Statistical analyses were performed with IBM SPSS Statistics for Macintosh, Version 28 [61]. Participant demographics, trauma histories, and symptoms were evaluated with chi-square tests, Pearson’s correlations, and independent sample t-tests for differences across imaging sites. Linear regressions covarying for MRI scanner site, age, and sex at birth assessed effects of childhood maltreatment load and posttraumatic outcomes on FA in bilateral white matter tracts. These tests were conducted for the 18 individual white matter tracts included in the JHU atlas. FA was examined due to its predominance in the literature. Relationships with AD, RD, and MD were examined in exploratory follow-up analyses for significant tracts in the FA analysis (see Supplementary information). Identical follow-up tests evaluated the contribution of the subcomponents of tracts significantly associated with childhood maltreatment load. A nominal significance threshold was set at *p* < 0.05, 2-tailed. False discovery rate (FDR) correction using the Benjamini–Hochberg method was used to control for multiple comparisons and maintain *α* = 0.05. For statistically significant models where subcomponent data was available (e.g., the anterior limb of the internal capsule), identical follow-up models were completed with separate FDR correction using the Benjamini–Hochberg method. Linear models covarying for MRI scanner site, age, and sex at birth evaluated effects of summed exposure to moderate to extreme threat (physical, emotional, and sexual abuse) and deprivation (emotional and physical neglect) components of childhood maltreatment load, as well as their interaction, on major bilateral white matter tracts significantly associated with childhood maltreatment load. Tracts that showed a significant association with childhood trauma were also included in subsequent mediation analyses, conducted using the PROCESS macro version 4 [62], including childhood trauma load, posttraumatic outcomes at 6-months, and a mediator of white matter microstructure. For mediation analyses, we completed bootstrapping with 5000 permutations to obtain 95% bias-corrected confidence intervals as an inferential test of direct and indirect effects. Lastly, univariate effects of childhood maltreatment load on 6-month bilateral white matter tracts significantly associated with childhood maltreatment at 2-weeks were evaluated with ANOVA, in models covarying for scanner site, age, and sex assigned at birth.

## Results

### Participant characteristics

Participant demographics and trauma characteristics are detailed in Table 1. Samples from the imaging sites were well matched across sex assigned at birth, age, educational attainment, employment, total family income, and marital status (Table S2). Further, each MRI scanning site sample had similar distributions of participants’ qualifying traumas and proportions of individuals who hit their head as part of the trauma (Supplementary Information). Participant racial/ethnic identity significantly differed by site (*p* < 0.001).

**Table 1:** Demographics and trauma characteristics.

		Overall (*n* = 202)
Sex Assigned at Birth, *N* (%)	Male	72 (35.6)
	Female	130 (64.4)
Age, M (SD)	–	35.4 (12.99)
Highest Grade, *N* (%)	Some HS/Below	9 (4.5)
	High School/GED	52 (25.7)
	Some College	70 (34.7)
	Associate Degree	21 (10.4)
	Bachelor’s Degree	36 (17.8)
	Graduate Degree	14 (6.9)
Employment, *N* (%)	Employed	140 (69.3)
	Retired	6 (3)
	Homemaker	9 (4.5)
	Student	9 (4.5)
	Unemployed, Disabled/Other	38 (18.8)
Total Family Income (TFI), *N* (%)	TFI ≤$19000	54 (26.7)
	$19001≤TFI≤$35000	69 (34.2)
	$35001≤TFI≤$50000	28 (13.9)
	$50001≤TFI≤$75000	19 (9.4)
	$75001≤TFI≤$100000	12 (5.9)
	TFI*≥*$100001	19 (9.4)
Race-Ethnicity, *N* (%)	Hispanic	26 (12.9)
	Non-Hispanic White	72 (35.6)
	Non-Hispanic Black	92 (45.5)
	Non-Hispanic Other	11 (5.4)
Marital, *N* (%)	Married	36 (17.8)
	Separated	3 (1.5)
	Divorced	27 (13.4)
	Widowed	2 (1.0)
	Never Married	134 (65.8)
ED Event, *N* (%)	Motor Vehicle Collision	151 (74.8)
	Physical Assault	18 (8.9)
	Sexual Assault	1 (.5)
	Fall > = 10 feet	3 (1.5)
	Non-motor Collision	8 (4.0)
	Fall <10 / unknown	9 (4.5)
	Burns	1 (.5)
	Animal Related	7 (3.5)
	Other	4 (2)
Hit Head, *N* (%)	No	98 (48.5)
	Yes	86 (42.6)
	Missing	18 (8.9)

*ED* Emergency Department.

Childhood and lifetime trauma load among participants are detailed in Table 2 and distribution of childhood maltreatment load scores in Table S3. On average, participants endorsed greater than one moderate to extreme childhood maltreatment type, with emotional abuse (32.7%) being the most frequently endorsed followed by sexual abuse (24.8%) and emotional neglect (24.8%). There were no significant site differences in the prevalence of any maltreatment subtype or the average number of moderate to extreme maltreatment subtypes endorsed (Table S4). However, there were significant site differences in the modified total LEC score (*p* = 0.002). Associations between childhood maltreatment load, modified total LEC score, and 6-month symptom scores at each site are shown in Table S5. Participants did not significantly differ in 6-month PTSD, depression, anxiety, or dissociation symptoms across sites (Table S6). Of note, participants included in the present analyses reported significantly lower 6-month PTSD symptoms and total childhood trauma scores than those excluded due to MRI issues but did not differ in childhood maltreatment load (Supplementary Information).

**Table 2:** Childhood and lifetime trauma load.

		Overall (*n* = 202)
# Mlx Types (0–5), M (SD)	–	1.21 (1.53)
Emotional Abuse, M (SD)		2.50 (2.63)
Mod-Extreme Emotional Abuse, *N* (%) / M (SD)	No	136 (67.3)
	Yes	66 (32.7)
Sexual Abuse, M (SD)		1.66 (2.91)
Mod-Extreme Sexual Abuse, *N* (%) / M (SD)	No	152 (75.2)
	Yes	50 (24.8)
Physical Abuse, M (SD)		1.56 (2.46)
Mod-Extreme Physical Abuse, *N* (%) / M (SD)	No	158 (78.2)
	Yes	44 (21.8)
Emotional Neglect, M (SD)		1.86 (2.36)
Mod-Extreme Emotional Neglect, *N* (%) / M (SD)	No	152 (75.2)
	Yes	50 (24.8)
Physical Neglect, M (SD)		1.49 (2.15)
Mod-Extreme Physical Neglect, *N* (%) / M (SD)	No	168 (83.2)
	Yes	34 (16.8)
CTQ Total (0–44), M (SD)	–	9.91 (9.91)
mLEC-5 Score (0–17), M (SD)		6.77 (4.65)

Note: *Mlx* Maltreatment; *CTQ* Childhood Trauma Questionnaire (11 item); *mLEC-5 Score* modified total Life Events Checklist Score.

### Childhood maltreatment and white matter

**Fig. 1:**
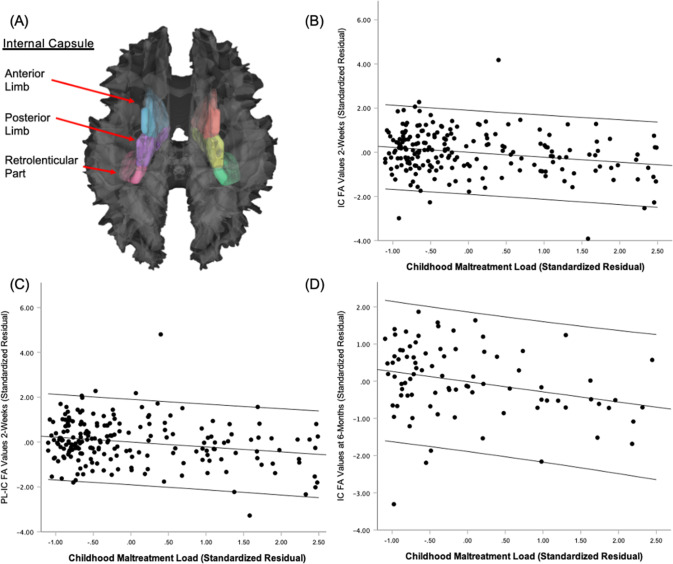
The relationship between childhood maltreatment load and internal capsule FA values at 2-weeks and 6-months. The internal capsule and its subcomponents are displayed on 3D rendering of human white matter tracts (**A**) Standardized residual plot of the regression of Childhood Maltreatment Load on 2-Week Internal Capsule FA Values depicts the significant negative effect (**B**). Standardized residual plot of Childhood Maltreatment Load on 2-Week Posterior-Limb of the Internal Capsule FA Values depicts the significant negative effect (**C**). Standardized residual plot of the regression of Childhood Maltreatment Load on Internal Capsule FA Values indexed 6-months post-trauma depicts the significant negative effect (**D**).

Childhood maltreatment load was associated with FA of several white matter tracts (Table 3). Following FDR correction, childhood maltreatment load negatively varied with FA in bilateral internal capsule (IC) at 2-weeks post-trauma, after covarying for sex assigned at birth, scanner site, and age (Table 3). Given the significant relationship between the IC and childhood maltreatment load, we considered the contribution of the IC subcomponents including the Posterior Limb of the IC (PL-IC), the Retrolenticular Part of the IC (RL-IC), and the Anterior Limb of the IC (AL-IC) in identical models and found childhood maltreatment load significantly negatively varied with all 3 IC subcomponents after FDR correction, though the PL-IC was the strongest contributor to the effect (Table 3; Fig. 1). Exploratory follow-up analyses with other diffusivity metrics revealed childhood maltreatment load was also associated with RD in the PL-IC (Table S7). Additional statistical analyses found only the threat (physical, emotional, and sexual abuse; β = −0.19, *p* = 0.01), not deprivation (emotional and physical neglect; β = −0.05, *p* = 0.53), component of childhood maltreatment load significantly contributed to the observed effect on the IC when both dimensions were included in an identical model as described above. The interaction between threat and deprivation was not significant. We further conducted sensitivity analyses to determine if associations between IC FA and childhood maltreatment remained while controlling for prior (i.e., endorsed pre-trauma) PCL-5 scores or mLEC-5 scores. Inclusion of either covariate did not impact the relationship between IC FA and childhood maltreatment load (see Supplementary information).

**Table 3:** Significant univariate effects in childhood maltreatment load model (2-Week).

	β	t-statistic	FDR adjusted *p*-values
IC	−0.21	−3.195	0.029**
*—PL-IC*	−0.21	−3.114	0.006**
*—RL-IC*	−0.18	−2.655	0.013**
*—AL-IC*	−0.13	−2.237	0.026**
CR	−0.14	−2.276	0.215
G-CC	−0.12	−2.155	0.194
SS	−0.14	−2.001	0.211

Reported tracts were significant at a nominal *p* < 0.05. Benjamini–Hochberg adjusted *p*-values were calculated by multiplying raw *p*-values by *m/i* (i = rank, *m* = total number of tests). Internal Capsule subcomponents were included in a follow-up, identical model, and FDR corrected with Benjamini–Hochberg. The PL-IC, RL-IC, and AL-IC were tested in follow-up models given the significant IC effect and separately FDR corrected. **Significant After Benjamini–Hochberg (*p* < 0.05) Adjustment.

*IC* Internal Capsule, *CR* Corona Radiata, *G-CC* Genu of the Corpus Callosum, *SS* Sagittal Stratum, *PL-IC* Posterior Limb of the Internal Capsule, *RL-IC* Retrolenticular Part of Internal Capsule, *AL-IC* Anterior Limb of Internal Capsule.

We performed follow-up analyses to test whether associations between childhood maltreatment and FA of the IC were also observed at 6-months post-trauma. Childhood maltreatment load negatively predicted bilateral IC FA indexed 6-months after trauma (Table 4; Fig. 1). In further analyses of IC subparts, negative predictive relationships of childhood maltreatment load with PL-IC and AL-IC microstructure were significant following Benjamini–Hochberg FDR correction (Table 4).

**Table 4:** Univariate effects in childhood maltreatment load model (6-Month).

	β	t-statistic	FDR adjusted *p*-value
IC	−0.28	−2.552	0.026**
—PL-IC	−0.31	−2.946	0.016**
—AL-IC	−0.24	−2.318	0.031**
—RL-IC	−0.21	−1.889	0.063

Benjamini–Hochberg adjusted *p*-values were calculated by multiplying raw *p*-values by *m/i* (i = rank, *m* = total number of tests). Internal Capsule subcomponents were included in a follow-up, identical model. The Benjamini–Hochberg FDR-correction included all 6-month tests. **Significant After Benjamini–Hochberg (*p* < 0.05) Adjustment.

*PL-IC* Posterior Limb of the Internal Capsule, *IC* Internal Capsule, *AL-IC* Anterior Limb of Internal Capsule, *RL-IC* Retrolenticular Part of Internal Capsule.

### Mediation analyses: childhood maltreatment, IC microstructure, and 6-month PTSD symptoms

**Fig. 2:**
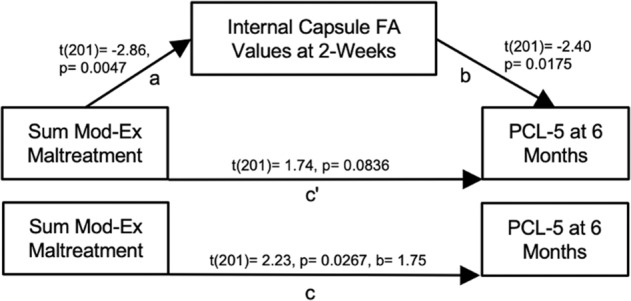
Mediation model of the effect of childhood maltreatment load on 6-month PCL-5 through Internal Capsule FA values indexed 2-weeks post-trauma. The indirect effect is significant based on a 5000 permutation, bootstrapped 95% confidence interval (i.e., path ab; b = 0.37, Boot SE = 0.18, 95% CI = [0.05, 0.76]), completely mediating the effect of childhood maltreatment load on PCL-5 scores (i.e., path c'; b = 1.37, SE = 0.79, 95% CI = [−0.18, 2.93]).

Mediation analyses revealed a total effect of childhood maltreatment load on PCL-5 scores at 6-months (b = 1.75, SE = 0.78. 95% CI = [0.20, 3.29]). We found a significant indirect effect of childhood maltreatment load on 6-month PCL-5 scores through IC microstructure (b = 0.37, Boot SE = 0.18, 95% CI = [0.05, 0.76]) that completely mediated the effect of childhood maltreatment load on PCL-5 scores (b = 1.37, SE = 0.79, 95% CI = [−0.18, 2.93]) (Fig. 2). Similar analyses were performed with the total childhood maltreatment score, and we observed similar results (see Supplementary information).

Exploratory mediation analyses assessed if findings were specific to future PTSD symptoms or if similar relationships were observed with other posttraumatic outcomes including depression, anxiety, and dissociation (Supplementary Information; Figure S1). Although there was a total effect of childhood maltreatment load on 6-month PROMIS-Depression (b = 1.29, SE = 0.48, 95% CI = [0.34, 2.24]), the indirect effect of childhood maltreatment load on 6-month PROMIS-Depression through IC microstructure (b = 0.15, Boot SE = 0.12, 95% CI = [−0.07, 0.39]) was not significant and did not mediate the effect of childhood maltreatment load on 6-month PROMIS-Depression scores (b = 1.14, SE = 0.49, 95% CI = [0.17, 2.11]). There was a total effect of childhood maltreatment load on 6-month PROMIS-Anxiety (b = 0.43, SE = 0.20, 95% CI = [0.05, 0.82]); however, neither the indirect effect of childhood maltreatment load on 6-month PROMIS-Anxiety through IC microstructure (b = 0.05, Boot SE = 0.05, 95% CI = [−0.04, 0.14]) nor the direct effect of childhood maltreatment load on 6-month PROMIS-Anxiety (b = 0.39, SE = 0.20, 95% CI = [−0.01, 0.78]) met statistical significance. No total effect of childhood maltreatment load on dissociation emerged (b = 0.14, SE = 0.08, 95% CI = [−0.01, 0.29]).

## Discussion

To our knowledge, the present study is the first to investigate the relationship between childhood maltreatment load and white matter microstructure with posttraumatic symptoms in the early aftermath of trauma. We observed robust relationships between childhood maltreatment load and fractional anisotropy (FA) in the internal capsule (IC) in the early aftermath of an acute trauma event (2-weeks) and 6-months later. Furthermore, variations in IC FA values at 2-weeks fully mediated the relationship between childhood maltreatment load and later posttraumatic symptoms at 6-months. The mediation was specific to posttraumatic symptoms and not observed for depressive, anxiety, or dissociative symptomatology. Given that childhood maltreatment was related to IC microstructure at 2-weeks and 6-months following the adulthood traumatic event, our findings may point to IC FA values as a stable biomarker of later posttraumatic dysfunction and suggest a potential neurobiological pathway through which childhood trauma could confer risk for acute stress reactions in adulthood. Additionally, this study did not reproduce effects of white matter tracts previously found to vary with PTSD symptoms and childhood trauma exposure, including the cingulum bundle, uncinate fasciculus, and corpus callosum.

Our findings implicate the IC as a critical substrate for the effects of childhood trauma on PTSD development. The IC is a dense fiber bundle that contains several projections including the corticospinal tract, frontopontine and corticofugal fibers, the anterior and superior thalamic radiation, the optic radiation, and the auditory radiation [63, 64]. Anatomically, the IC is limited laterally by the pallidum and medially by the thalamus, the head of the caudate nucleus, and the corticospinal tract [65]. The IC further appears to contain fibers for both medial (hippocampal formation, mammillary bodies, anterior thalamic nuclei, and cingulate gyrus) and basolateral (orbitofrontal cortex, dorsomedial thalamic nucleus, amygdala, and anterior temporal cortex) limbic circuits [66].

The present findings may be related to dysfunctional stimulus processing during PTSD. Although threat processing and its neural substrates are commonly dysregulated in PTSD, these components are dependent on the ability to perceive and integrate sensory stimuli. Recent work suggests variability in structure of visual processing regions, such as the ventral visual stream, is associated with susceptibility to PTSD symptom development [36, 67]. This pathway supports important processes, such as object recognition, integral to threat learning and includes core threat-related regions such as the amygdala and medial PFC [68]. In the current study, we found that higher childhood maltreatment load was associated with lower FA of the IC and its subcomponents. The IC encompasses occipital connections between the higher order visual cortex and temporal lobe [64], as well as components of major motor tracts and somatosensory relays from the thalamus to the cortex [63]. Prior work found trauma-exposed children and adults with childhood maltreatment histories had reduced FA of the IC and its component tracts, including the optic radiations and left anterior thalamic radiation [25, 69]. Speculatively, reduced FA of the IC may reflect disrupted white matter myelination and membrane integrity in fibers that transmit visual sensory information and contribute to altered perception and processing of threat-related information, which, in turn, may contribute to PTSD-related disruptions. Disruption of the IC could further be related to altered ability to consolidate, encode, or retrieve sensory components of trauma memories leading emotion dysregulation. In line with such reasoning, ischemic damage to the IC can lead to cognitive and behavioral alterations such as agitation and impaired attention [63], and deep brain stimulation of the ventral IC/ventral striatum enhances prefrontal cortex driven cognitive control [70]. However, corresponding data on visual processing was not collected in the present study, and specific interpretations of IC function should thus be tempered. Taken together with prior literature, the present results suggest childhood maltreatment has a pronounced effect on IC microstructure which may confer risk for PTSD-related dysregulation following subsequent trauma.

Of note, we did not observe effects in canonical threat circuitry often associated with PTSD. Past studies have not typically considered childhood maltreatment when evaluating white matter markers of PTSD susceptibility [15–18], and it is possible that reduced IC FA may be a sequela of childhood maltreatment exposure. Moreover, although we analyzed imaging data from over 200 participants, we could have been underpowered to detect all effects with our unbiased whole-brain analytic approach. There are likely different biological subtypes of PTSD that are not accounted for here and such heterogeneity may have decreased our ability to detect associations in other tracts. For example, subtypes that show stronger intrusive symptoms or disruptions in emotional memory may be more associated with canonical threat neurocircuitry. Notably, varied white matter microstructure in the IC and its component tracts has been previously implicated in PTSD [11], with recent works suggesting a role in predicting PTSD in the acute aftermath of trauma exposure [16] and in treatment response [71]. Further investigation of the role of childhood maltreatment load in the relationship between white matter microstructure and PTSD development might assist in developing robust predictive models.

The findings of the current work should be interpreted with several considerations. First, we assessed childhood maltreatment load with a retrospective self-assessment. Since we did not query the age participants experienced childhood trauma, we could not assess the role of the developmental timing of trauma on the observed effects. Future longitudinal work is needed to investigate white matter microstructural variability in children and recently traumatized adults with more granular information on childhood trauma exposure and timing. Further, although we used items from the childhood trauma questionnaire, which is itself a validated and broadly used tool, and prospective research suggests the reliability of such retrospective reporting [40, 72–74], we were unable to administer the full questionnaire as to minimize participant burden within the parent study. It would also be beneficial to investigate these associations in longitudinal studies of childhood trauma as opposed to using purely retrospective reports. Secondly, data that could be related to hypothesized contributions of the IC to sensory processes were not available, and thus the specific functional role of variability within IC microstructure in relation to PTSD is unclear. Future research considering the specific targets and functional outcomes of variable IC microstructure among those with childhood trauma would further clarify the present findings. Lastly, our analyses do not consider potential protective or socioeconomic factors that may contribute to early life stress load or resiliency. We did, however, consider the site at which MRI scanning occurred, which largely accounted for participant race and ethnicity. Given the relationships between racial discrimination, neighborhood disadvantage, and socioeconomic status with white matter microstructure [75–77], a critical next step will be to understand how these factors and protective agents impact white matter markers of PTSD susceptibility. As participants in this work reported substantial childhood maltreatment and low levels of PTSD symptoms prior to the presenting trauma, resiliency factors may be especially critical to decipher. Relatedly, it will also be important to consider potentially salient factors such as prenatal exposures and genetics.

The present study of recent trauma survivors examined the relationship of childhood maltreatment load with white matter microstructure and posttraumatic symptoms in the early aftermath of trauma. Childhood maltreatment load stably, inversely varied with the FA of the IC following the acute trauma event. Further, the FA of the IC in the early aftermath of an acute trauma event mediated the relationship between childhood maltreatment load and PTSD symptoms 6-months following the adulthood trauma exposure. These findings suggest a unique role for IC microstructure as a neural pathway between childhood trauma and future PTSD symptoms following a recent trauma. Furthermore, these data suggest DTI imaging may assist in revealing neural signatures of risk for later stress-related dysfunction in those with earlier childhood trauma.

### Supplementary information

Supplementary material
