# Supplementary material for: Internal capsule microstructure mediates the relationship between childhood maltreatment and PTSD following adulthood trauma exposure
Source: Mol Psychiatry. 2023 Mar 17;28(12):5140–9. doi: 10.1038/s41380-023-02012-3 (PMC10505244; doi:10.1038/s41380-023-02012-3)
Supplement: Supplementary file 1 — Supplementary material [file 41380_2023_2012_MOESM1_ESM.docx]

**Supplementary Material to “Internal capsule microstructure mediates the relationship between childhood maltreatment and PTSD following adulthood trauma exposure”**

1. **Supplementary Results**

**1.1 Internal reliability of childhood maltreatment items**

Cronbach’s alpha was calculated on the 11 selected items from the CTQ-SF to estimate internal reliability of the composite measure of childhood maltreatment. In the subsample of AURORA participants with MRI data used in the present analyses (n = 202), the items showed a Cronbach’s alpha of a = 0.92. The alpha value is similar to that observed in the initial sample of survey data (i.e., those who provided usable CTQ-SF item data) from the parent study within the same timeframe (a = 0.91). Thus, the childhood maltreatment items selected for the present study showed high internal reliability.

**1.2 Mediation models of Internal Capsule (IC) subparts**

Exploratory analyses of FA values of the IC subparts revealed a significant indirect effect of childhood maltreatment load on 6-month PCL-5 scores through retrolenticular limb of the IC (RL-IC) FA values (b=0.30, Boot SE=0.17, 95% CI=[0.01, 0.68]) that completely mediated the effect of childhood maltreatment load on PCL-5 scores (b=1.45, SE=0.78, 95% CI=[-0.09, 3.00]). No significant indirect effects through anterior limb of the IC FA values (AL-IC; b=0.21, Boot SE=0.13, 95% CI=[-0.02, 0.50]) or posterior limb of the IC FA values (PL-IC; b=0.27, Boot SE=0.18, 95% CI=[-0.05, 0.64]) emerged. However, the direct effect of childhood maltreatment load on 6-month PCL-5 scores was not significant after controlling for the indirect path through AL-IC (b=1.53, SE=0.78, 95% CI=[-0.01, 3.08]) and PLIC (b=1.47, SE=0.80, 95% CI=[-0.09, 3.04]) FA values.

**1.3 Differences in maltreatment and symptoms among those included and excluded in imaging analysis**

We compared childhood maltreatment load and posttraumatic symptoms of those excluded in imaging quality control or missing one self-report measure to those included in the present analyses. Included and excluded groups did not differ in childhood maltreatment load (*t(*293)=1.86, *p*=0.065), however the groups did differ in total childhood trauma scores (*t*(293)=2.34, *p*=.0.20) and 6-month PCL-5 scores (*t*(247)=3.72, *p*=.001) with greater childhood trauma (M_diff_ = 2.95) and PCL-5 (M_diff_ = 10.47) scores in the excluded groups.

**1.4 Secondary analyses using total childhood maltreatment scores in analyses of Internal Capsule (IC) fractional anisotropy (FA).**

Our main analyses focused on childhood maltreatment load as a function of the number of moderate to severe exposures of each type. We performed secondary analyses of IC FA using the total score from items used to quantify childhood maltreatment. Childhood maltreatment total scores were negatively related to IC FA (*t*(194) = -3.251, p = 0.001). In mediation models, we observed a significant indirect effect of childhood maltreatment total scores on 6-month PCL-5 scores through the IC FA (b=0.05, SE=0.03, 95% CI=[0.03, 0.12]). However, the direct effect of childhood maltreatment load on 6-month PCL-5 scores was also significant after controlling for the indirect path (b=0.31, SE=0.12, 95% CI=[0.07, 0.55]). Thus, compared to childhood maltreatment load, usage of total scores does not result in a full mediation of the effect of childhood maltreatment on 6-month PCL-5 scores.

**1.5 Sensitivity analyses of association between childhood maltreat and white matter microstructure of the Internal Capsule (IC) covarying for prior PCL-5 scores or modified LEC-5 scores.**

Given variability in PTSD symptoms endorsed before the traumatic event, we conducted sensitivity analyses between IC FA and childhood maltreatment load including an additional covariate of PCL-5 scores reported as present prior to trauma. Pre-trauma PCL-5 scores (t(131) = -1.59, p = 0.113) and mLEC-5 scores (t(181) = 0.47, p = 0.637) were not associated with IC FA values. Childhood maltreatment load was associated with IC FA values (t(131) = -2.22, p = 0.03) when covarying for PCL-5 scores or mLEC-5 scores (t(181) = -2.95, p = 0.004). These data suggest the association between IC microstructure and childhood maltreatment load is robust with regards to earlier PTSD symptoms.

**1.6 Radial Diffusivity (RD), Axial Diffusivity (AD), and Mean Diffusivity (MD) of the Internal Capsule (IC) and subparts**

Linear regressions covarying for MRI scanner site, age, and sex at birth assessed effects of childhood maltreatment load on RD, AD, and MD of the IC and its subparts and are reported in table S6. In mediation models, no significant indirect effects of childhood maltreatment load on 6-month PCL-5 scores through the RD (b=0.17, SE=0.12, 95% CI=[-0.09, 0.42]), AD (b=0.00, SE=0.10, 95% CI=[-0.26, 016]), or MD (b=0.04, SE=0.09, 95% CI=[-0.21, 0.19]) of the IC emerged. However, the direct effect of childhood maltreatment load on 6-month PCL-5 scores was not significant after controlling for the indirect path through RD of the IC (b=1.54, SE=0.79, 95% CI=[-0.01, 3.10]).

**Table S1. Site specific scanner information**

|  | Site1  Siemens TIM 3T Trio  (12 Channel Head Coil) | Site2  Siemens TIM 3T Trio  (12 Channel Head Coil) | Site3  Siemens MAGNETOM 3T Prisma  (20 Channel Head Coil) | Site4  Siemens 3T Verio  (12 Channel Head Coil) | Site5  Siemens MAGNETOM 3T Prisma  (20 Channel Head Coil) |
| --- | --- | --- | --- | --- | --- |
| Modality |  |  |  |  |  |
| T1-weighted | **TR** = 2530ms, **TEs** = 1.74/3.6/5.46/7.32ms, **TI** = 1260ms, **flip angle** = 7, **FOV** = 256mm, **slices** = 176, **Voxel size** = 1mm x 1mm x 1mm | **TR** = 2530ms, **TEs** = 1.74/3.6/5.46/7.32ms, **TI** = 1260ms, **flip angle** = 7, **FOV** = 256mm, **slices** = 176, **Voxel size** = 1mm x 1mm x 1mm | **TR** = 2300ms, **TE** = 2.96ms, **TI** = 900ms, **flip angle** = 9, **FOV** = 256mm, **slices** = 176, **Voxel size** = 1.2mm x 1.0mm x 12mm | **TR** = 2530ms, **TEs** = 1.74/3.65/5.51/7.72ms, **TI** = 1260ms, **flip angle** = 7, **FOV** = 256mm, **slices** = 176, **Voxel size** = 1mm x 1mm x 1mm | **TR** = 2300ms, **TE** = 2.98ms, **TI** = 900ms, **flip angle** = 9, **FOV** = 256mm, **slices** = 176, **Voxel size** = 1.2mm x 1.0mm x 12mm |
| Diffusion Weighted Imaging | **TR** = 7700ms, **TE** = 85ms, **FOV** = 212mm, **flip angle** = 90, **Volumes** = 71 (64 **b**=1000 s/mm^2,^ 7 b0), **PA-encoded**, **Voxel size** = 2mm x 2mm x 2mm | **TR** = 7700ms, **TE** = 85ms, **FOV** = 212mm, **flip angle** = 90, **Volumes** = 71 (64 **b**=1000 s/mm^2,^ 7 b0), **PA-encoded**, **Voxel size** = 2mm x 2mm x 2mm | **TR** = 7000ms, **TE** = 74ms, **FOV** = 212mm, **flip angle** = 90, **Volumes** = 71 (64 **b**=1000 s/mm^2,^ 7 b0), **PA-encoded**, **Voxel size** = 2mm x 2mm x 2mm | **TR** = 12000ms, **TE** = 85ms, **FOV** = 212mm, **flip angle** = 90, **Volumes** = 71 (64 **b**=1000 s/mm^2,^ 7 b0), **PA-encoded**, **Voxel size** = 2mm x 2mm x 2mm | **TR** = 7700ms, **TE** = 67ms, **FOV** = 212mm, **flip angle** = 90, **Volumes** = 71 (64 **b**=1000 s/mm^2,^ 7 b0), **PA-encoded**, **Voxel size** = 2mm x 2mm x 2mm |

**Table S2. Demographics and trauma characteristics by scanner site**

|  |  | Overall (n=202) | McLean (n=72) | WUSL (n=14) | WSU (n=50) | Temple (n=59) | Emory (n=7) | p-value |
| --- | --- | --- | --- | --- | --- | --- | --- | --- |
| Sex Assigned at Birth,  N (%) | Male  Female | 72 (35.6)  130 (64.4) | 27 (37.5)  45 (62.5) | 4 (28.6)  10 (71.4) | 21 (42)  29 (58) | 17 (28.8)  42 (71.2) | 3 (42.9)  4 (57.1) | 0.618 (χ^2^) |
| Age, M (SD) | --- | 35.4 (12.99) | 34.76 (12.85) | 42.29 (13.11) | 35.66 (13.23) | 35.41 (13.18) | 29.29 (7.41) | 0.226 (ANOVA) |
| Highest Grade, N (%) | Some HS/Below  High School/GED  Some College  Associate Degree  Bachelor’s Degree  Graduate Degree | 9 (4.5)  52 (25.7)  70 (34.7)  21 (10.4)  36 (17.8)  14 (6.9) | 1 (1.4)  23 (31.9)  20 (27.8)  11 (15.3)  12 (16.7)  5 (6.9) | 1 (7.1)  4 (28.6)  6 (42.9)  1 (7.1)  2 (14.3)  --- | 3 (6)  9 (18)  19 (38)  5 (10)  9 (18)  5 (10) | 4 (6.8)  14 (23.7)  24 (40.7)  4 (6.8)  9 (15.3)  4 (6.8) | ---  2 (28.6)  1 (14.3)  ---  4 (57.1)  -- | 0.448 (χ^2^) |
| Employment, N (%) | Employed  Retired  Homemaker  Student  Unemployed, Disabled/Other | 140 (69.3)  6 (3)  9 (4.5)  9 (4.5)  38 (18.8) | 50 (69.4)  1 (1.4)  2 (2.8)  4 (5.6)  15 (20.8) | 11 (78.6)  ---  ---  ---  3 (21.4) | 36 (72)  2 (4)  3 (6)  2 (4)  7 (14) | 38 (64.4)  3 (5.1)  4 (6.8)  3 (5.1)  11 (18.6) | 5 (71.4)  ---  ---  ---  2 (28.6) | 0.963 (χ^2^) |
| Total Family Income (TFI), N (%) | TFI ≤$19000  $19001≤TFI≤$35000  $35001≤TFI≤$50000  $50001≤TFI≤$75000  $75001≤TFI≤$100000  TFI$\geq$$100001 | 54 (26.7)  69 (34.2)  28 (13.9)  19 (9.4)  12 (5.9)  19 (9.4) | 19 (26.4)  24 (33.3)  7 (9.7)  7 (9.7)  8 (11.1)  6 (8.3) | 4 (28.6)  4 (28.6)  3 (21.4)  2 (14.3)  ----  1 (7.1) | 6 (12)  20 (40)  11 (22)  3 (6)  2 (4)  8 (18) | 23 (39.0)  18 (30.5)  6 (10.2)  6 (10.2)  2 (3.4)  4 (6.8) | 2 (28.6)  3 (42.9)  1 (14.3)  1 (14.3)  ---  --- | 0.412 (χ^2^) |
| Race-Ethnicity, N (%) | Hispanic  Non-Hispanic White  Non-Hispanic Black  Non-Hispanic Other | 26 (12.9)  72 (35.6)  92 (45.5)  11 (5.4) | 18 (25.0)  30 (41.7)  18 (25.0)  6 (8.3) | ----  4 (28.6)  10 (71.4)  ---- | 4 (8)  20 (40)  26 (52)  ---- | 3 (5.1)  17 (28.8)  33 (55.9)  5 (8.5) | 1 (14.3)  1 (14.3)  5 (71.4)  ---- | <.001 (χ^2^) |
| Marital, N (%) | Married  Separated  Divorced  Widowed  Never Married | 36 (17.8)  3 (1.5)  27 (13.4)  2 (1.0)  134 (65.8) | 9 (12.3)  1 (1.4)  13 (17.8)  ----  49 (68.5) | 5 (35.7)  1 (7.1)  1 (7.1)  ----  7 (50.0) | 9 (18)  9 (18)  2 (4)  ---  29 (58) | 12 (20.3)  1 (1.7)  4 (6.8)  ----  42 (71.2) | 1 (14.3)  ---  ---  --  6 (85.7) | 0.183 (χ^2^) |
| ED Event, N (%) | Motor Vehicle C  Physical Assault  Sexual Assault  Fall >= 10 feet  Non-motor Collision  Fall <10 / unknown  Burns  Animal Related  Other | 151 (74.8)  18 (8.9)  1 (.5)  3 (1.5)  8 (4.0)  9 (4.5)  1 (.5)  7 (3.5)  4 (2) | 55 (76.4)  6 (8.3)  1 (1.4)  2 (2.8)  4 (5.6)  3 (4.2)  ---  1 (1.4)  --- | 11 (78.6)  1 (7.1)  ---  ---  ---  1 (7.1)  ---  ---  1 (7.1) | 32 (64)  5 (10)  ---  ---  2 (4)  4 (8)  ---  5 (10)  2 (4) | 47 (79.7)  6 (10.2)  ---  ---  2 (3.4)  1 (1.7)  1 (1.7)  1 (1.7)  1 (1.7) | 6 (85.7)  ---  ---  1 (14.3)  ---  ---  ---  ---  --- | 0.384 (χ^2^) |
| Hit Head, N (%) | 0  1  Missing | 98 (48.5)  86 (42.6)  18 (8.9) | 35 (48.6)  31 (43.1)  6 (8.3) | 6 (42.9)  6 (42.9)  2 (14.3) | 26 (52)  21 (42)  3 (6) | 28 (47.5)  26 (44.1)  5 (8.5) | 3 (42.9)  2 (28.6)  2 (28.6) | 0.795 (χ^2^) |

**Table S3. Frequency of scores for the maltreatment load.**

| Maltreatment Load Score | Frequency (n) | Percentage (%) |
| --- | --- | --- |
| 0 | 99 | 49.01 |
| 1 | 39 | 19.31 |
| 2 | 19 | 9.41 |
| 3 | 23 | 11.39 |
| 4 | 12 | 5.94 |
| 5 | 10 | 4.95 |

**Table S4.** **Childhood and lifetime trauma load across study sites.**

|  |  | Overall (n=202) | McLean (n=72) | WUSL (n=14) | WSU (n=50) | Temple (n=59) | Emory (n=7) | p-value |
| --- | --- | --- | --- | --- | --- | --- | --- | --- |
| **# Mlx Types (0-5),** M (SD) | --- | 1.21 (1.53) | 1.40 (1.60) | .64 (1.15) | 1.10 (1.49) | 1.24 (1.56) | .86 (1.46) | 0.447 (ANOVA) |
| **Mod-Extreme Emotional Abuse,** N (%) | No  Yes | 136 (67.3)  66 (32.7) | 47 (65.3)  25 (34.7) | 11 (78.6)  3 (21.4) | 32 (64)  18 (36) | 40 (67.8)  19 (32.2) | 6 (87.5)  1 (14.3) | 0.685 (χ^2^) |
| **Mod-Extreme Sexual Abuse,** N (%) | No  Yes | 152 (75.2)  50 (24.8) | 49 (68.1)  23 (31.5) | 11 (78.6)  3 (21.4) | 40 (80)  10 (20) | 47 (79.7)  12 (20.3) | 5 (71.4)  2 (28.6) | 0.499 (χ^2^) |
| **Mod-Extreme Physical Abuse,** N (%) | No  Yes | 158 (78.2)  44 (21.8) | 56 (77.8)  16 (22.2) | 11 (78.6)  3 (21.4) | 42 (84)  8 (16) | 43 (72.9)  16 (27.1) | 6 (85.7)  1 (14.3) | 0.698 (χ^2^) |
| **Mod-Extreme Emotional Neglect,** N (%) | No  Yes | 152 (75.2)  50 (24.8) | 51 (70.8)  21 (29.2) | 14 (100)  ---- | 39 (78)  11 (22) | 42 (71.2)  17 (28.8) | 6 (85.7)  1 (14.3) | 0.165 (χ^2^) |
| **Mod-Extreme Physical Neglect,** N (%) | No  Yes | 168 (83.2)  34 (16.8) | 56.8 (77.8)  16 (22.2) | 14 (100)  ---- | 42 (84)  8 (16) | 50 (84.7)  9 (15.3) | 6 (85.7)  1 (14.3) | 0.344 (χ^2^) |
| **CTQ Total (0-44),** M (SD) | --- | 9.91 (9.911) | 9.89 (9.89) | 5.57 (6.836) | 8.56 (10.02) | 9.46 (10.49) | 8.00 (10.31) | 0.641 (ANOVA) |
| **mLEC-5 Score (0-17)**, M (SD) |  | 6.77 (4.65) | 7.53 (4.84) | 5.71 (4.05) | 6.54 (4.30) | 5.54 (4.18) | 12.42 (5.29) | 0.002 (ANOVA) |

**Table S5.** **Correlations of maltreatment load with lifetime trauma and posttraumatic outcomes across study sites.**

|  | **Overall (n=202)** | | **McLean (n=72)** | | | **WUSL (n=14)** | | | **WSU (n=50)** | | | **Temple (n=59)** | | | **Emory (n=7)** | | |
| --- | --- | --- | --- | --- | --- | --- | --- | --- | --- | --- | --- | --- | --- | --- | --- | --- | --- |
|  | r | p | r | | p | r | | p | r | p | | | r | p | r | p | |
| **# Mlx Types** |  | |  | | |  | | |  | | | |  | |  | | |
| Modified Total LEC-5 Score | 0.18 | 0.015 | 0.21 | 0.084 | | -0.04 | 0.892 | | 0.06 | | 0.677 | | 0.30 | .030 | -0.06 | | 0.906 |
| 6-Month PCL-5 | 0.16 | 0.027 | 0.06 | 0.613 | | -0.30 | 0.301 | | 0.39 | | 0.005 | | 0.29 | 0.028 | -0.09 | | 0.843 |
| 6-Month PROMIS-Dep | 0.19 | 0.008 | 0.04 | 0.716 | | -0.23 | 0.441 | | 0.37 | | 0.009 | | 0.29 | 0.026 | 0.38 | | 0.400 |
| 6-Month PROMIS-Anx | 0.16 | 0.028 | 0.05 | 0.700 | | -0.27 | 0.382 | | 0.21 | | 0.140 | | 0.31 | 0.016 | 0.36 | | 0.427 |
| 6-Month DES-B Modified | -0.13 | 0.067 | 0.06 | 0.637 | | 0.08 | 0.791 | | 0.13 | | 0.359 | | 0.25 | 0.060 | -0.08 | | 0.860 |
| **CTQ Total** |  | |  | | |  | | |  | | | |  | |  | | |
| Modified Total LEC Score | 0.22 | 0.003 | 0.22 | | 0.076 | -0.14 | 0.624 | | 0.17 | | 0.253 | | 0.37 | 0.006 | -0.07 | | 0.881 |
| 6-Month PCL-5 | 0.21 | 0.002 | 0.10, | | 0.422 | -0.23 | 0.424 | | 0.48 | | <0.001 | | 0.34 | 0.009 | -0.04 | | 0.940 |
| 6-Month PROMIS-Dep | 0.23 | 0.001 | -0.07 | | 0.550 | -0.01 | 0.983 | | 0.43 | | 0.002 | | 0.29 | 0.024 | 0.46 | | 0.298 |
| 6-Month PROMIS-Anx | 0.21 | 0.004 | -0.10 | | 0.421 | -0.11, | 0.714 | | 0.24 | | 0.099 | | 0.34 | 0.009 | 0.44 | | 0.324 |
| 6-Month DES-B Modified | -0.16 | 0.021 | 0.10 | | 0.414 | 0.12 | 0.686 | | 0.19 | | 0.191 | | 0.26 | 0.051 | -0.04 | | 0.930 |

Modified LEC-5 total score n=190 and post traumatic outcomes n=198. WUSL: n=13 for PROMIS-D, PROMIS-A, and DES-B. WSU: Modified LEC total score n=48 , and n=49 for PROMIS-D, PROMIS-A, and DES-B. Temple: Modified LEC total score n=53.

**Table S6. 6-Month posttraumatic outcomes by scanner site**

|  | Overall (n=198) | McLean (n=72) | WUSL (n=14) | WSU (n=50) | Temple (n=59) | Emory (n=7) | p-value |
| --- | --- | --- | --- | --- | --- | --- | --- |
| **PCL-5 Total Score** M (SD) | 20.72 (17.12) | 19.89 (18.64) | 26.79 (18.64) | 18.56 (11.73) | 22.22 (19.09) | 20.00 (12.29) | .520 |
| **PROMIS-Depression T Score** M (SD) | 51.62 (10.40) | 50.98 (10.65) | 53.29 (8.89) | 52.59 (9.07) | 51.48 (11.69) | 49.40 (9.25) | .854 |
| **PROMIS-Anxiety Total Score** M (SD) | 5.75 (4.20) | 5.35 (4.20) | 6.54 (4.48) | 5.78 (3.48) | 6.03 (4.73) | 5.86 (4.28) | .852 |
| **DES-B Modified Total Score** M (SD) | 1.11 (1.64) | 1.15 (1.80) | 1.62 (1.26) | 1.00 (1.35) | 1.10 (1.78) | 0.43 (0.79) | .608 |

**Table S7.** *Univariate effects of childhood maltreatment load on axial diffusivity, radial diffusivity, and mean diffusivity of the internal capsule*

|  |  | **RD** |  |  | **AD** |  |  | **MD** |  |
| --- | --- | --- | --- | --- | --- | --- | --- | --- | --- |
|  | **β** | **t-statistic** | **p** | **β** | **t-statistic** | **p** | **β** | **t-statistic** | **p** |
| **IC** | 0.14 | 2.186 | 0.030* | -0.07 | -1.086 | 0.279 | 0.06 | 0.999 | 0.319 |
| *—PL-IC* | 0.15 | 2.317 | 0.022* | -0.08 | -1.316 | 0.190 | 0.05 | 0.793 | 0.429 |
| *—RL-IC* | 0.12 | 1.738 | 0.084 | -0.06 | -0.881 | 0.379 | 0.06 | 0.872 | 0.384 |
| *—AL-IC* | 0.11 | 1.826 | 0.069 | -0.03 | -0.553 | 0.581 | 0.06 | 1.039 | 0.300 |

*Significant at a threshold of p<0.05. Note: RD = Radial Diffusivity; AD = Axial Diffusivity; MD = Mean diffusivity; IC = internal Capsule; PL-IC = Posterior Limb of the Internal Capsule; IC = Internal Capsule; RL-IC = Retrolenticular Part of Internal Capsule; AL-IC = Anterior Limb of Internal Capsule.

**Figure S1. Mediation models of symptom severity.** The indirect effect of childhood maltreatment load on later depression through IC FA values was not significant based on a 5000 permutation, bootstrapped 95% confidence interval (b=0.15, Boot SE=0.12, 95% CI=[-0.07, 0.39]). The indirect effect of childhood maltreatment load on later anxiety through IC FA values was not significant based on a 5000 permutation, bootstrapped 95% confidence interval (b=0.05, Boot SE=0.05, 95% CI=[-0.04, 0.14]). No total effect of childhood maltreatment load on dissociation emerged (b=0.14, SE=0.08, 95% CI=[-0.01, 0.29]).

**
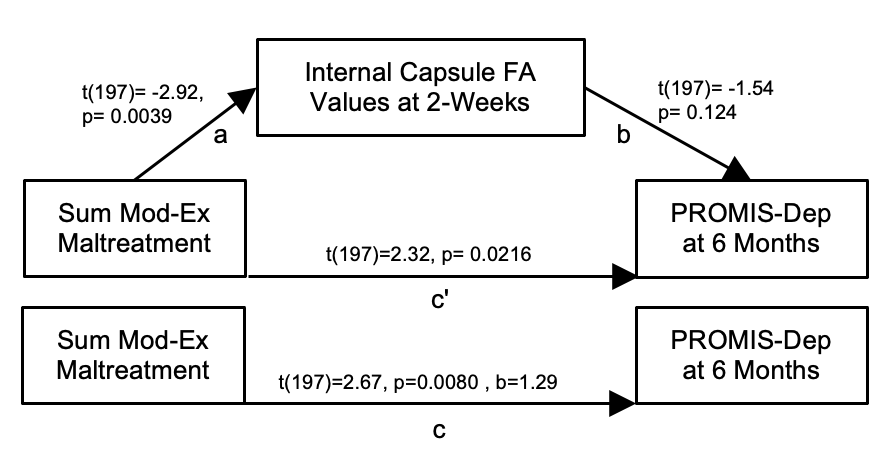

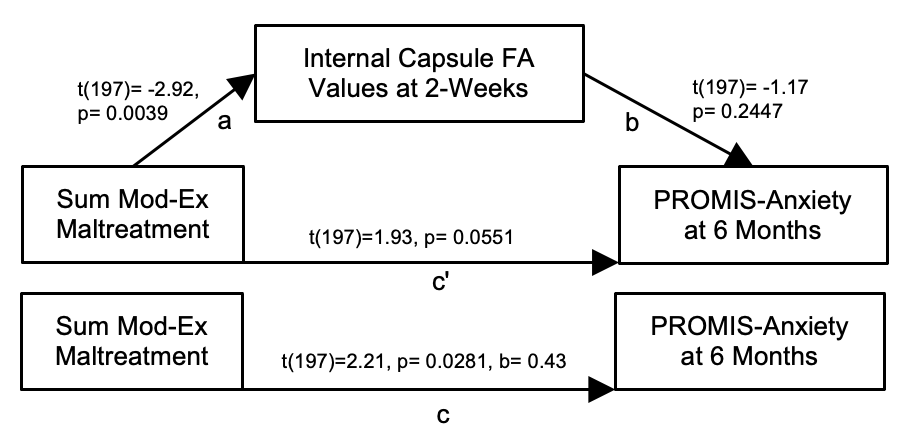
**

**
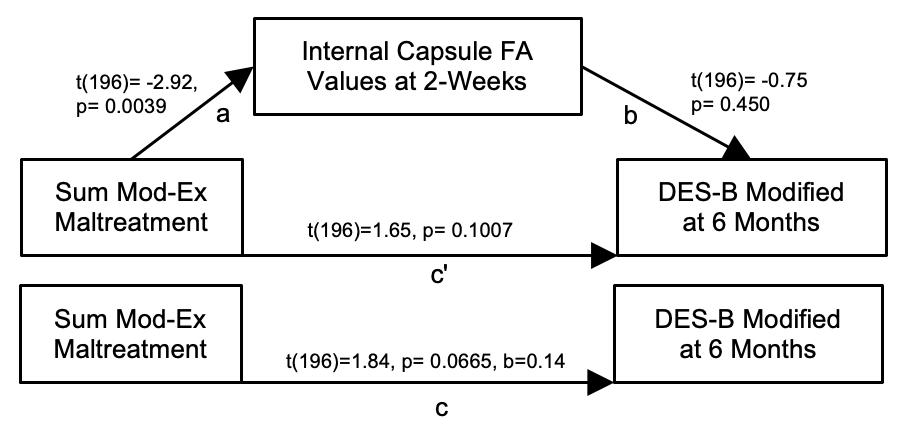
**

**Appendix A. Questions from the Childhood Trauma Questionnaire in the present analysis**

Emotional Abuse:

- Prompt - “And how often did you have each of the following experiences during your childhood?”
  - Question 1 “You were emotionally abused.”
  - Question 2 “People in your family said hurtful or insulting things about you.”

Physical Abuse:

- Prompt - “And how often did you have each of the following experiences during your childhood?”
  - Question 1 “People in your family hit you so hard that it left you with bruises or marks.”
  - Question 2 “You were physically abused.”

Sexual Abuse:

- Prompt - “And how often did you have each of the following experiences during your childhood?”
  - Question 1 “Someone tried to make you do sexual things or watch sexual things.”
  - Question 2 “Someone molested you.”
  - Question 3 “You were sexually abused.”

Emotional Neglect:

- Prompt - “And how often did you have each of the following experiences during your childhood?”
  - Question 1 “There was someone in your family who helped you feel that you were important or special.”
  - Question 2 “You felt loved.”

Physical Neglect:

- Prompt - “And how often did you have each of the following experiences during your childhood?”
  - Question 1 “You knew there was someone to take care of you and protect you.”
  - Question 2 “There was someone to take you to the doctor if you needed it.”
